# Season of birth and the risk of type 2 diabetes in adulthood: a prospective cohort study of 0.5 million Chinese adults

**DOI:** 10.1007/s00125-016-4200-4

**Published:** 2017-01-07

**Authors:** Jiahui Si, Canqing Yu, Yu Guo, Zheng Bian, Xia Li, Ling Yang, Yiping Chen, Huarong Sun, Bo Yu, Junshi Chen, Zhengming Chen, Jun Lv, Liming Li, on behalf of the China Kadoorie Biobank Collaborative Group

**Affiliations:** 1 Department of Epidemiology and Biostatistics, School of Public Health, Peking University Health Science Center, 38 Xueyuan Road, Beijing, 100191 People’s Republic of China; 2 Chinese Academy of Medical Sciences, Beijing, People’s Republic of China; 3 Clinical Trial Service Unit & Epidemiological Studies Unit (CTSU), Nuffield Department of Population Health, University of Oxford, Oxford, UK; 4 NCDs Prevention and Control Department, Huixian Center for Disease Control and Prevention, Xinxiang, Henan People’s Republic of China; 5 NCDs Prevention and Control Department, Nangang Center for Disease Control and Prevention, Harbin, Heilongjiang People’s Republic of China; 6 China National Center for Food Safety Risk Assessment, Beijing, People’s Republic of China; 7 Peking University Institute of Environmental Medicine, Beijing, People’s Republic of China

**Keywords:** Fetal development, Seasons, Type 2 diabetes

## Abstract

**Aims/hypothesis:**

Season of birth as a surrogate for potential environmental exposure during fetal development and early postnatal life has shown an inconsistent association with adult type 2 diabetes in white populations living in high-latitude regions. The present study aimed to examine the association between birth seasonality and risk of adult type 2 diabetes in Chinese individuals living across wide regions of low latitude and lower to middle latitude.

**Methods:**

Participants from the China Kadoorie Biobank were enrolled during 2004–2008 and followed up until 31 December 2013. After excluding participants with cancer, heart disease, stroke and diabetes at baseline, the present study included 189,153 men and 272,058 women aged 30–79 years. We used multivariable Cox proportional hazards model to estimate the HR and 95% CI.

**Results:**

During a median follow-up of 7.2 years (3.3 million person-years), we documented 8784 incident cases of type 2 diabetes. In the whole cohort, compared with summer-born participants, the adjusted HRs (95% CIs) were 1.09 (1.02, 1.16), 1.08 (1.02, 1.15) and 1.09 (1.02, 1.15) for those who were born in Spring, Autumn and Winter, respectively. The association was consistent in both men and women and across subgroups defined by residence and lifestyle factors later in life.

**Conclusions/interpretation:**

In this large prospective study, participants born in summer had a lower risk of adult type 2 diabetes compared with other seasons of birth, suggesting exposures in early life with some degree of seasonal variation might influence the risk of adult diabetes.

**Electronic supplementary material:**

The online version of this article (doi:10.1007/s00125-016-4200-4) contains peer-reviewed but unedited supplementary material, which is available to authorised users.

## Introduction

In 2014, there were 387 million people living with diabetes and 4.9 million deaths attributable to diabetes worldwide [1]. In China, the rapidly increasing prevalence of diabetes reached 9.7% in 2010 [2]. Type 2 diabetes represents about 85–95% of all cases of diabetes [1]. There is a growing interest in the contribution of prenatal environmental exposure to the risk of type 2 diabetes in adulthood. It has been suggested that adverse environmental influences during critical periods of prenatal growth could permanently change the structure and function of organs and tissues, leading to increased susceptibility to the development of type 2 diabetes in later life [3].

In addition to special famine events, the season or month of birth often serves as a surrogate for potential environmental exposure during perinatal life. Factors exhibiting seasonal variation include, but are not limited to, exposure to sunlight, food availability and eating habits, and outdoor physical activity [4–8]. Only a few studies conducted in white populations living in relatively high-latitude regions have examined the association between the season or month of birth and type 2 diabetes in adulthood, with mixed results [9–11].

In the present study, we prospectively examined the association between season of birth and risk of type 2 diabetes in adulthood in the China Kadoorie Biobank (CKB; see the electronic supplementary material [ESM] for a list of members of the CKB Collaborative Group) study of 0.5 million adults, a population living across wide regions of low latitude and lower to middle latitude. We additionally assessed whether several lifestyle factors later in life might modify the association between early life exposures and later risk of type 2 diabetes.

## Methods

### Study population

Further details of the CKB have been given elsewhere [12, 13]. Briefly, a total of 512,891 participants aged 30–79 years were recruited in 2004–2008 from ten geographically diverse survey sites across China: five urban sites (Harbin, Qingdao, Suzhou, Liuzhou and Haikou) and five rural sites (Henan, Gansu, Sichuan, Zhejiang and Hunan). The enrolment rate of the population was comparable across latitudes and between urban or rural sites, and almost one in three (33% in rural areas and 27% in urban areas) responded. The study protocol was approved by the Ethics Review Committee of the Chinese Center for Disease Control and Prevention (Beijing, China) and the Oxford Tropical Research Ethics Committee, University of Oxford (UK). All participants provided written informed consent before taking part in the study.

In the present analysis, participants with major chronic diseases at baseline were excluded due to potential changes in lifestyle and subsequent risk of diabetes. We excluded participants who reported medical histories of heart disease (*n* = 15,472), stroke (*n* = 8884) or cancer (*n* = 2577), or had prevalent diabetes (*n* = 30,300) based on self-reported or glucose testing at baseline (fasting glucose of 7.0 mmol/l or more, or random glucose of 11.1 mmol/l or more). Such exclusion was conducted to prevent an incidence/prevalence bias and rule out the possibility of reverse causation for adjusted covariates including lifestyle and adiposity measures, and diabetes. We also excluded three people who were lost to follow-up shortly after baseline and two people with missing BMI data. After these exclusions, 461,211 participants remained for the final analyses.

### Assessment of exposure and covariates

All participants reported their date of birth at baseline. We categorised the month of birth into Spring (March, April and May), Summer (June, July and August), Autumn (September, October and November) and Winter (December, January and February).

Covariate information was obtained from a baseline questionnaire; this included sociodemographic status (age, sex, education and marital status), lifestyle behaviours (tobacco smoking, alcohol consumption, physical activity and intakes of fresh vegetables, fruit and red meat), body weight at 25 years of age, family history of diabetes and women’s menopausal status.

Trained staff measured body weight, standing height, sitting height and waist circumference (WC) using a standard protocol and calibrated instruments. BMI was calculated as weight in kilograms divided by the square of the standing height in metres. Weight change since 25 years of age was calculated as the difference between measured weight at baseline and weight at 25 years old. The length of the leg was calculated as the difference between standing and sitting height.

### Ascertainment of incident type 2 diabetes

Incident cases of type 2 diabetes were identified by linkage with local disease and death registries, with the recently established national health insurance (HI) system and by active follow-up (i.e. visiting local communities or directly contacting participants). The electronic linkage with the HI claim databases is one of the most important means of ascertaining incident cases of diabetes and was achieved for 95% of the participants in 2013. Both urban and rural participants and different latitudes of sites had similar proportions of successful linkage to HI databases. For the present analysis, we included diabetes cases coded by the ICD-10 (www.who.int/classifications/icd/en/) as E11 and E14. Other cases clearly defined as non-type 2 diabetes were excluded. Although misclassification from other types of diabetes may exist, the number of individuals with any non-type 2 diabetes was small as the majority of our participants were aged over 40 years. The diagnostic validity of incident diabetes was adjudicated in a random sample of 831 reported cases from all ten survey sites, with a review of hospital medical records. Overall, 98.6% of diagnoses of diabetes were confirmed.

### Statistical analysis

Participants contributed person-time data from baseline until the date of diagnosis of diabetes, death, loss to follow-up or 31 December 2013, whichever came first. We used multivariable Cox proportional hazards model to estimate the HR and 95% CI, with age as the underlying time scale, and stratified by 5-year age groups (age at baseline) and ten survey sites.

Multivariable models for association between season or month of birth and risk of diabetes in adulthood were adjusted for: age (years); sex (male or female; for whole cohort); level of education (no formal schooling, primary school, middle school, high school, college, or university or higher); marital status (married, widowed, divorced or separated, or never married); alcohol consumption (not weekly drinker, weekly but not daily drinker, daily drinker with an intake <15, 15–29, 30–59 or ≥60 g/day); smoking status (never or occasional smoker, former smoker having quit smoking ≥5 or <5 years previously, or current daily smoker smoking <15, 15–24 or ≥25 cigarettes per day); physical activity (metabolic equivalent of task [MET] × h/day); frequencies of intake of red meat, fresh fruit and vegetables (daily, 4–6 days/week, 1–3 days/week, monthly, or rarely or never); family history of diabetes (presence, absence or unknown); and menopausal status (premenopausal, perimenopausal or postmenopausal; for women only). We further explored whether the association between season of birth and risk of diabetes in adulthood was confounded or mediated by BMI at baseline. We additionally adjusted for weight gain since 25 years of age as a sensitivity analysis. To control for potential confounding effects of early life factors, we additionally adjusted for leg length, a biomarker of early life conditions [14, 15]. The risk estimates did not change materially (data not shown).

We also conducted analyses stratified according to prespecified baseline subgroups: residence (urban or rural sites), latitude (seven sites in middle latitudes, or three sites in low latitudes, with 30 degrees north latitude as the dividing line), age at baseline (<50, 50–59 or ≥60 years), smoking status (daily smoker or not), alcohol consumption (weekly drinker or not), level of physical activity (categorised using tertile cutoffs), BMI (<24.0, 24.0–27.9 or ≥28.0 kg/m^2^), WC (central obesity, with men ≥ 85 cm and women ≥80 cm, or not), and weight gain since 25 years of age (<2.5, 2.5–9.9 or ≥10.0 kg).

We used Stata version 14.0 (StataCorp, College Station, TX, USA) to analyse the data. Statistical significance was set at two-tailed *p* < 0.05.

## Results

Of all participants, 41.0% (*n* = 189,153) were men, and 57.8% (*n* = 266,352) resided in rural areas. Table 1 presents the baseline characteristics of the participants according to the season of birth. Participants who were born in Spring and Summer had higher measures of adiposity and a larger increase in weight since 25 years.

**Table 1:** Baseline characteristics according to the season of birth among 461,211 participants

Characteristics	Spring	Summer	Autumn	Winter
No. of participants	105,779	112,042	128,548	114,842
Age (years)	50.4 (10.4)	50.7 (10.4)^†^	50.8 (10.5)^†^	51.0 (10.6)
Rural area (%)	58.7^†^	57.7	56.5	58.3^†^
Married (%)	91.1^†^	91.2^†^	91.2^†^	90.9^†^
Middle school and higher (%)	49.6^†‡§^	48.9	49.3^†‡^	49.7^†§^
Daily smoker (%)	26.9^†^	27.1^†^	27.1^†^	26.9^†^
Weekly drinker (%)	15.2^†^	15.3^†^	15.3^†^	15.0^†^
Physical activity (MET × h/day)*	21.9 (13.9)^†^	21.9 (14.0)^†^	21.8 (14.0)^‡^	21.8 (13.9)^†‡^
Weekly consumption^a^				
Red meat (day)	3.69 (2.54)^†‡§^	3.69 (2.53)^†‡^	3.71 (2.52)^†‡§^	3.71 (2.51)^†§^
Fresh vegetables (day)	6.83 (0.83)^†^	6.84 (0.78)^†^	6.84 (0.77)^†^	6.84 (0.75)^†^
Fresh fruit (day)	2.56 (2.48)^†^	2.55 (2.46)^†^	2.56 (2.48)^†^	2.56 (2.47)^†^
Postmenopausal (%)^b^	48.8	49.2^†^	49.4^†^	49.2^†^
Family history of diabetes (%)	9.2^†^	9.1^†^	9.0^†^	9.2^†^
BMI (kg/m^2^)	23.58 (3.35)^†^	23.55 (3.35)^†^	23.50 (3.30)^‡^	23.48 (3.31)^‡^
WC (cm)	80.0 (9.6)	79.8 (9.6)	79.6 (9.5)^†^	79.6 (9.6)^†^
Weight change since 25 years of age (kg)^c^	4.8 (8.9)^†^	4.8 (8.9)^†^	4.6 (8.8)^‡^	4.5 (8.9)^‡^

Values are mean (SD) unless otherwise stated. All variables were adjusted for age, sex and survey sites, as appropriate

^a^Weekly consumptions of red meat, fresh vegetables and fruit were calculated by assigning participants to the midpoint of their consumption category

^b^Among 272,059 female participants

^c^ *n* = 386,753

^†‡§^Percentages or means sharing the same symbol were not significantly different at a Bonferroni-corrected level of significance. The Bonferroni-adjusted threshold of significance was 0.008, based on six tests (pairwise comparison between four birth seasons)

**Fig. 1:**
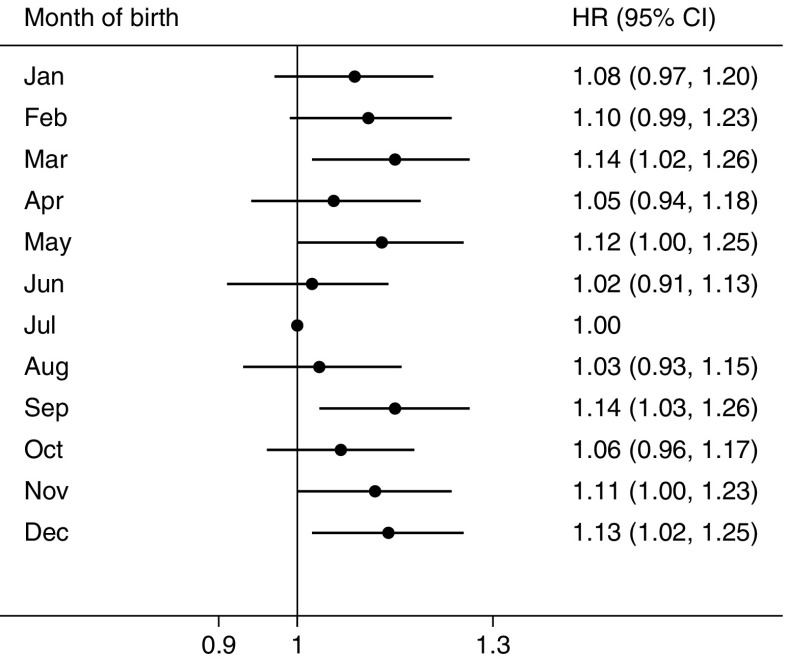
HRs and 95% CIs for incident type 2 diabetes in adulthood according to month of birth. Horizontal lines represent 95% CIs. July is the reference month, so CIs are not presented. The multivariable model was adjusted for age, sex, level of education, marital status, alcohol consumption, smoking status, physical activity, intake frequencies of red meat, fresh fruit and vegetables, family history of diabetes and BMI at baseline

During a median of 7.2 years (interquartile range 1.88 years, 3.3 million person-years) of follow-up, we documented 3259 incident cases of type 2 diabetes among men and 5525 cases among women. In multivariable-adjusted models, the season of birth was significantly associated with the risk of incident type 2 diabetes. Further adjustment for BMI at baseline did not materially change the association. In all eligible participants, compared with participants who were born in Summer, the adjusted HRs (95% CIs) for the risk of type 2 diabetes in adulthood were 1.09 (1.02,1.16), 1.08 (1.02, 1.15) and 1.09 (1.02, 1.15) for those who were born in Spring, Autumn and Winter, respectively (Table 2). The combined HR (95% CI) for seasons of birth other than Summer was 1.09 (1.03, 1.14); the respective HRs (95% CIs) were 1.11 (1.02, 1.20) among men and 1.07 (1.01, 1.14) among women. No statistically significant difference between men and women was observed in the association between season of birth and risk of type 2 diabetes (*p* = 0.670 for interaction between four seasons of birth and sex; *p* = 0.704 for interaction between two groups of seasons of birth and sex). Figure 1 further presents the association between month of birth and risk of diabetes. Despite a small fluctuation in some individual months, we observed a similar seasonal variation, with participants born in Summer having the lowest risk of diabetes in adulthood.

**Table 2:** HR (95% CI) for incident type 2 diabetes by the season of birth among 461,211 participants

Variable	Spring	Summer	Autumn	Winter	Combined category of Spring, Autumn and Winter^a^
Whole cohort					
No. of person-years	753,382	800,360	918,565	819,588	2,491,535
No. of cases	1971	1996	2523	2294	6788
Age adjusted	1.09 (1.02, 1.16)	1.00	1.07 (1.01, 1.14)	1.07 (1.01, 1.14)	1.08 (1.02, 1.13)
Multivariable adjusted^b^	1.09 (1.02, 1.16)	1.00	1.07 (1.01, 1.14)	1.07 (1.01, 1.14)	1.08 (1.02, 1.13)
+BMI at baseline	1.09 (1.02, 1.16)	1.00	1.08 (1.02, 1.15)	1.09 (1.02, 1.15)	1.09 (1.03, 1.14)
Men					
No. of person-years	301,255	323,106	375,124	337,313	1,013,691
No. of cases	727	721	966	845	2538
Age adjusted	1.11 (1.00, 1.23)	1.00	1.12 (1.02, 1.24)	1.07 (0.97, 1.18)	1.10 (1.01, 1.19)
Multivariable adjusted^b^	1.10 (0.99, 1.22)	1.00	1.12 (1.02, 1.23)	1.06 (0.96, 1.18)	1.10 (1.01, 1.19)
+BMI at baseline	1.10 (0.99, 1.22)	1.00	1.13 (1.03, 1.25)	1.08 (0.98, 1.20)	1.11 (1.02, 1.20)
Women					
No. of person-years	452,127	477,255	543,441	482,275	1,477,844
No. of cases	1244	1275	1557	1449	4250
Age adjusted	1.08 (1.00, 1.17)	1.00	1.05 (0.97, 1.13)	1.07 (0.99, 1.16)	1.07 (1.00, 1.13)
Multivariable adjusted^b^	1.08 (1.00, 1.17)	1.00	1.05 (0.98, 1.13)	1.07 (1.00, 1.16)	1.07 (1.00, 1.14)
+BMI at baseline	1.08 (1.00, 1.17)	1.00	1.06 (0.98, 1.14)	1.09 (1.01, 1.17)	1.07 (1.01, 1.14)

^a^Reference group: Summer-born participants

^b^Multivariable model was adjusted for: age (years); sex (male or female; for whole cohort); level of education (no formal schooling, primary school, middle school, high school, college, or university or higher); marital status (married, widowed, divorced or separated, or never married); alcohol consumption (not weekly drinker, weekly but not daily drinker, daily drinker with an intake of <15, 15–29, 30–59 or ≥60 g/day); smoking status (never or occasional smoker, former smoker having quit smoking ≥5 or <5 years previously, or current daily smoker smoking <15, 15–24 or ≥25 cigarettes per day); physical activity (MET × h/day); intake frequencies of red meat, fresh fruit and vegetables (daily, 4–6 days/week, 1–3 days/week, monthly, or rarely or never); family history of diabetes (presence, absence or unknown); and menopausal status (premenopausal, perimenopausal or postmenopausal; for women only)

**Fig. 2:**
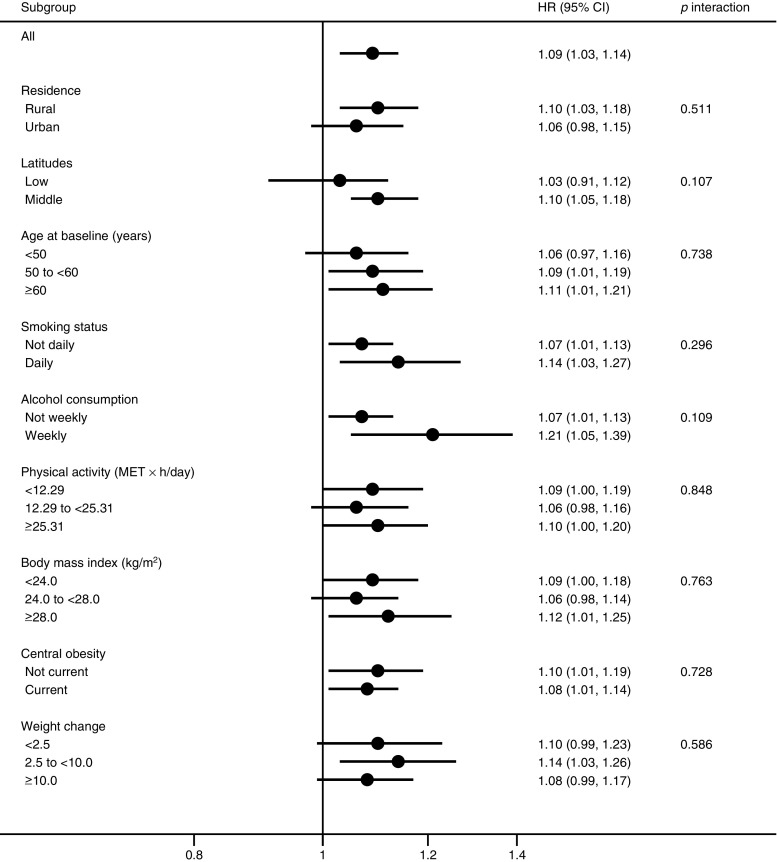
Subgroup analysis of the association between season of birth and risk of type 2 diabetes in adulthood among the whole cohort according to potential baseline risk factors. Horizontal lines represent 95% CIs. HRs and 95% CIs are for comparison of a combined category of Spring-, Autumn- and Winter-born participants with Summer-born participants. The multivariable model was adjusted for age, sex, level of education, marital status, alcohol consumption, smoking status, physical activity, intake frequencies of red meat, fresh fruit and vegetables, family history of diabetes and BMI at baseline. The tests for interaction were performed using likelihood ratio tests, which involved comparing models with and without cross-product terms between the baseline stratifying variable and the season of birth. Subgroup analysis according to weight change since 25 years of age: *n* = 386,753

In subgroup analyses, we further examined the consistency of the association between season of birth and risk of diabetes among different subpopulations defined by multiple baseline characteristics of the participants. The positive association was generally similar across subgroups stratified according to rural/urban residence, residence at different latitudes, age, smoking status, alcohol consumption, level of physical activity, BMI, WC and weight gain since 25 years of age (all *p* values >0.05 for interaction) (Fig. 2 and ESM Table 1).

## Discussion

In this large prospective cohort of Chinese adults, we found that seasons of birth other than Summer were associated with an increased risk of type 2 diabetes in adulthood. Compared with Summer-born participants, those born in other seasons had a 9% higher risk of diabetes. The association was consistent in both men and women and across subgroups defined by residence and lifestyle factors later in life.

To our knowledge, only three previous studies conducted in European populations have examined the association between birth seasonality and risk of type 2 diabetes in adulthood, and have shown inconsistent results. Findings from a Dutch hospital-based series study of 282 patients aged 30–90 years with type 2 diabetes showed an excess of diabetes births in the first quarter of the year and a deficiency in the final quarter when comparing the month of birth with the standard birth curve [9]. In another study conducted in three regions of Ukraine, a variation in season of birth was observed in 52,214 individuals with type 2 diabetes who were born before 1960, with a peak in April and a nadir between November and December, compared with the month-of-birth patterns in the general population [10]. The only prospective study conducted in a Danish population-based cohort of 223,099 adults born between 1930 and 1989 showed no association between birth seasonality and risk of type 2 diabetes [11]. The present study has found different results from those reported above, especially from those of a Danish study with a comparable study design. The explanation for this difference in results is not clear and might include differences in population characteristics, geographical location and adjustment for potential confounding factors. For example, the Danish population resided at a relatively high latitude and experienced fewer seasonal variations, and therefore fewer variations in environmental factors, than our population.

The relation between season of birth and type 2 diabetes is inconsistent with our previous findings in the same population that showed a Spring and early Summer peak and a Winter trough for BMI and WC [16]. Low exposure to ultraviolet B light and subsequently reduced levels of vitamin D during the late second and early third trimesters have been suggested as an explanation for the association between season of birth and adult adiposity measures. In addition, in the present study, the risk estimates of type 2 diabetes did not materially change after adjusting for BMI at baseline or weight gain since the age 25 years, suggesting that adult adiposity did not mediate the effects of birth season on diabetes risk, and that other mechanisms might be at work.

Impaired fetal nutrition in late gestation has been linked to insulin resistance and type 2 diabetes in adulthood through permanent changes in the function of pancreatic beta cells or in the sensitivity of the tissues to insulin [17–19]. Although the nutritional challenge caused by seasonal variation is not as extreme as the one resulting from famine events, the variations in food variety, the nutritional value of food and eating habits across seasons were still evident a few decades ago, especially in rural regions of China. It is possible that Summer-born participants experienced a richer nutritional environment in their late prenatal period and showed a lower risk of type 2 diabetes in adulthood than those born in other seasons. The season or month of birth might also be indicative of some other factors across a time span from periconception to the early postnatal period. For example, mothers’ breastfeeding behaviour may vary in babies who are born in different seasons [20] and has also been linked to the risk of type 2 diabetes in the offspring [21]. However, this cannot explain what we observed in the CKB population. Further studies are warranted to explore the underlying mechanisms that link season of birth and type 2 diabetes in adulthood.

To the best of our knowledge, this is by far the largest prospective study assessing the association between season of birth and the risk of type 2 diabetes in adulthood. The present study was conducted across wide regions of low latitude and lower to middle latitude. This study acknowledges some limitations. We did not collect information about participants’ exposures and medical conditions during the prenatal and postnatal periods; this was because of a lack of documented perinatal health records for this generation of the Chinese population and because of potential recall bias for self-reported information. Confounding by unmeasured factors, such as birth weight, maternal nutrition during pregnancy, parental socioeconomic status and breastfeeding was still possible. In addition, the incident cases of diabetes in this study were mainly identified using linkage with the HI system. Thus underestimation of the incidence of type 2 diabetes might have occurred because some asymptomatic cases of diabetes were missed. However, the non-differential outcome misclassification on the season of birth might result in an attenuation of the effect estimates. Covariate information such as lifestyle behaviours and body weight at 25 years of age was self-reported. A measurement error in potential confounders might result in residual confounding. We excluded prevalent cases of diabetes at baseline to prevent incidence/prevalence bias and rule out the possibility of reverse causation. However, we acknowledge that such exclusion might limit the generalisability of the results to early-onset type 2 diabetes.

### Conclusion

In this large prospective study of Chinese adults, we found that participants born in Summer had a lower risk of type 2 diabetes in adulthood compared with other seasons of birth. The results provide clues that exposures in early life with some degree of seasonal variation might influence the risk of diabetes in adulthood. Further studies are essential to validate our findings and clarify the underlying mechanisms.

## Electronic supplementary material

Below is the link to the electronic supplementary material.

ESM (PDF 231 kb)

## Data Availability

Details of how to access CKB data and details of the data release schedule are available from www.ckbiobank.org/site/Data+Access.

## Abbreviations

CKB: China Kadoorie Biobank
HI: Health insurance
MET: Metabolic equivalent of task
WC: Waist circumference
