# Supplementary material for: Season of birth and the risk of type 2 diabetes in adulthood: a prospective cohort study of 0.5 million Chinese adults
Source: Diabetologia. 2017 Jan 7;60(5):836–42. doi: 10.1007/s00125-016-4200-4 (PMC6521727; doi:10.1007/s00125-016-4200-4)
Supplement: Supplementary file 1 — (PDF 231 kb) [file 125_2016_4200_MOESM1_ESM.pdf]

ESM Table 1 Association of the season of birth with the risk of type 2 diabetes in adulthood by baseline subgroup.

Subgroups	Summer			Combined category of Spring, Autumn and Winter[a]					*p* value for interation[b]
	No. of person-years	No. of cases	HR	No. of person-years	No. of cases in spring-born	No. of cases in autumn-born	No. of cases in winter-born	HR(95%CI)	
All	800,360	1996	1.00	2,491,535	6788	6788	6788	1.09 (1.03, 1.14)	
Residence									0.511
Rural	467,065	1196	1.00	1,454,583	1210	1527	1462	1.10 (1.03, 1.18)	
Urban	333,295	800	1.00	1,036,952	761	996	832	1.06 (0.98, 1.15)	
Latitudes									0.107
Low	220,706	511	1.00	660,135	444	599	492	1.03 (0.91, 1.12)	
Middle	579,654	1485	1.00	1,831,400	1527	1924	1802	1.10 (1.05, 1.18)	
Age									0.738
< 50	394,964	650	1.00	1,220,310	649	752	635	1.06 (0.97, 1.16)	
50 to < 60	242,412	730	1.00	756,843	696	978	884	1.09 (1.01, 1.19)	
≥ 60	162,984	616	1.00	514,382	626	793	775	1.11 (1.01, 1.21)	
Smoking status									0.296
Not daily	586,458	1551	1.00	1,822,439	1494	1898	1771	1.07 (1.01, 1.13)	
Daily	213,902	445	1.00	669,096	477	625	523	1.14 (1.03, 1.27)	
Alcohol consumption									0.109
Not weekly	679,067	1752	1.00	2,111,185	1723	2139	2005	1.07 (1.01, 1.13)	
Weekly	121,293	244	1.00	380,350	248	384	289	1.21 (1.05, 1.39)	
Physical activity									0.848
< 12.29	242,381	666	1.00	747,413	639	866	771	1.09 (1.00, 1.19)	

Subgroups	Summer			Combined category of Spring, Autumn and Winter[a]					*p* value for interation[b]
	No. of person-years	No. of cases	HR	No. of person-years	No. of cases in spring-born	No. of cases in autumn-born	No. of cases in winter-born	HR(95%CI)	
12.29 to < 25.31	272,362	677	1.00	852,983	652	845	783	1.06 (0.98, 1.16)	
≥ 25.31	285,617	653	1.00	891,139	680	812	740	1.10 (1.00, 1.20)	
Body mass index									0.763
< 24.0	461,293	691	1.00	1,446,552	651	922	838	1.09 (1.00, 1.18)	
24.0 to < 28.0	259,920	868	1.00	805,592	852	1063	965	1.06 (0.98, 1.14)	
≥ 28.0	79,147	437	1.00	239,390	468	538	491	1.12 (1.01, 1.25)	
Central obesity									0.728
Not current	485,378	731	1.00	1,526,171	684	994	903	1.10 (1.01, 1.19)	
Current	314,982	1265	1.00	965,364	1287	1529	1391	1.08 (1.01, 1.14)	
Weight change[c]									0.586
< 2.5	279,756	436	1.00	885,473	415	573	554	1.10 (0.99,1.23)	
2.5 to < 10.0	214,921	480	1.00	674,833	484	652	594	1.14 (1.03,1.26)	
≥ 10.0	178,419	743	1.00	537,061	748	902	789	1.08 (0.99,1.17)	

[a]Reference group: Summer-born participants

[b]The P values for interaction were computed with the use of likelihood-ratio tests comparing models with and without cross-product terms between the baseline stratifying variable and birth season

[c]n=386,753

Multivariable model was adjusted for: age (years); sex (male or female); level of education (no formal schooling, primary school, middle school, high school, college, or university or higher); marital status (married, widowed, divorced or separated, or never married); alcohol consumption (not weekly drinker, weekly but not daily drinker, daily drinker with an intake of<15, 15–29, 30–59 or ≥60 g/day); smoking status (never or occasional smoker, former smoker having quit smoking ≥5 or <5 years previously, or current daily smoker smoking <15, 15–24 or ≥25 cigarettes per day); physical activity (MET; h/day); intake frequencies of red meat, fresh fruit and vegetables (daily, 4–6 days/week, 1–3 days/week, monthly, or rarely or never); and family history of diabetes (presence, absence or unknown)

ESM: list of study group members

Members of the China Kadoorie Biobank collaborative group:

International Steering Committee: Junshi Chen, Zhengming Chen (PI), Rory Collins, Liming Li (PI), Richard Peto.

International Co-ordinating Centre, Oxford: Daniel Avery, Ruth Boxall, Derrick Bennett, Yumei Chang, Yiping Chen, Zhengming Chen, Robert Clarke, Huaidong Du, Simon Gilbert, Alex Hacker, Michael Holmes, Andri Iona, Christiana Kartsonaki; Rene Kerosi, Ling Kong, Om Kurmi, Garry Lancaster, Sarah Lewington, Kuang Lin, John McDonnell, Winnie Mei, Iona Millwood, Qunhua Nie, Jayakrishnan Radhakrishnan, Sajjad Rafiq, Paul Ryder, Sam Sansome, Dan Schmidt, Paul Sherliker, Rajani Sohoni, Iain Turnbull, Robin Walters, Jenny Wang, Lin Wang, Ling Yang, Xiaoming Yang. National Co-ordinating Centre, Beijing: Zheng Bian, Ge Chen, Yu Guo, Can Hou, Jun Lv, Pei Pei, Shuzhen Qu, Yunlong Tan, Canqing Yu. 10 Regional Co-ordinating Centres: Qingdao CDC: Zengchang Pang, Ruqin Gao, Shaojie Wang, Yongmei Liu, Ranran Du, Yajing Zang, Liang Cheng, Xiaocao Tian, Hua Zhang. Licang CDC: Silu Lv, Junzheng Wang, Wei Hou. Heilongjiang Provincial CDC: Jiyuan Yin, Ge Jiang, Xue Zhou. Nangang CDC: Liqiu Yang, Hui He, Bo Yu, Yanjie Li, Huaiyi Mu, Qinai Xu, Meiling Dou, Jiaojiao Ren. Hainan Provincial CDC: Shanqing Wang, Ximin Hu, Hongmei Wang, Jinyan Chen, Yan Fu, Zhenwang Fu, Xiaohuan Wang. Meilan CDC: Min Weng, Xiangyang Zheng, Yilei Li, Huimei Li,Yanjun Wang. Jiangsu Provincial CDC: Ming Wu, Jinyi Zhou, Ran Tao, Jie Yang. Suzhou CDC: Chuanming Ni, Jun Zhang, Yihe Hu, Yan Lu, , Liangcai Ma, Aiyu Tang, Shuo Zhang, Jianrong Jin, Jingchao Liu. Guangxi Provincial CDC: Zhenzhu Tang, Naying Chen, Ying Huang. Liuzhou CDC: Mingqiang Li, Jinhuai Meng, Rong Pan, Qilian Jiang, Weiyuan Zhang, Yun Liu, Liuping Wei, Liyuan Zhou, Ningyu Chen, Hairong Guan. Sichuan Provincial CDC: Xianping Wu, Ningmei Zhang, Xiaofang Chen, Xuefeng Tang. Pengzhou CDC: Guojin Luo, Jianguo Li, Xiaofang Chen, Xunfu Zhong, Jiaqiu Liu, Qiang Sun. Gansu Provincial CDC: Pengfei Ge, Xiaolan Ren, Caixia Dong. Maiji CDC: Hui Zhang, Enke Mao, Xiaoping Wang, Tao Wang, Xi zhang. Henan Provincial CDC: Ding Zhang, Gang Zhou, Shixian Feng, Liang Chang, Lei Fan. Huixian CDC: Yulian Gao, Tianyou He, Huarong Sun, Pan He, Chen Hu, Qiannan Lv, Xukui Zhang. Zhejiang Provincial CDC: Min Yu, Ruying Hu, Hao Wang. Tongxiang CDC: Yijian Qian, Chunmei Wang, Kaixue Xie, Lingli Chen, Yidan Zhang, Dongxia Pan. Hunan Provincial CDC: Yuelong Huang, Biyun Chen, Li Yin, Donghui Jin, Huilin Liu, Zhongxi Fu, Qiaohua Xu. Liuyang CDC: Xin Xu, Hao Zhang, Youping Xiong, Huajun Long, Xianzhi Li, Libo Zhang, Zhe Qiu.
